# Investigation of intra-fractionated range guided adaptive proton therapy: I. On-line PET imaging and range measurement

**DOI:** 10.1088/1361-6560/ad56f4

**Published:** 2024-07-17

**Authors:** Dongxu Yang, Xiaorong R Zhu, Mingli Chen, Lin Ma, Xinyi Cheng, David R Grosshans, Weiguo Lu, Yiping Shao

**Affiliations:** 1 Department of Radiation Oncology, University of Texas Southwestern Medical Center, Dallas, TX 75057, United States of America; 2 Department of Radiation Physics, University of Texas MD Anderson Cancer Center, Houston, TX 77000, United States of America; 3 Department of Radiation Oncology, University of Texas MD Anderson Cancer Center, Houston, TX 77000, United States of America

**Keywords:** proton beam range, proton-induced activity range, on-line PET imaging, intra-fractionated beam range measurement

## Abstract

**Objective.:**

Develop a prototype on-line positron emission tomography (PET) scanner and evaluate its capability of on-line imaging and intra-fractionated proton-induced radioactivity range measurement.

**Approach.:**

Each detector consists of 32 × 32 array of 2 × 2 × 30 mm^3^ Lutetium–Yttrium Oxyorthosilicate scintillators with single-scintillator-end readout through a 20 × 20 array of 3 × 3 mm^2^ Silicon Photomultipliers. The PET can be configurated with a full-ring of 20 detectors for conventional PET imaging or a partial-ring of 18 detectors for on-line imaging and range measurement. All detector-level readout and processing electronics are attached to the backside of the system gantry and their output signals are transferred to a field-programable-gate-array based system electronics and data acquisition that can be placed 2 m away from the gantry. The PET imaging performance and radioactivity range measurement capability were evaluated by both the offline study that placed a radioactive source with known intensity and distribution within a phantom and the online study that irradiated a phantom with proton beams under different radiation and imaging conditions.

**Main results.:**

The PET has 32 cm diameter and 6.5 cm axial length field-of-view (FOV), *~*2.3–5.0 mm spatial resolution within FOV, 3% sensitivity at the FOV center, 18%–30% energy resolution, and ~9 ns coincidence time resolution. The offline study shows the PET can determine the shift of distal falloff edge position of a known radioactivity distribution with the accuracy of 0.3 ± 0.3 mm even without attenuation and scatter corrections, and online study shows the PET can measure the shift of proton-induced positron radioactive range with the accuracy of 0.6 ± 0.3 mm from the data acquired with a short-acquisition (60 s) and low-dose (5 MU) proton radiation to a human head phantom.

**Significance.:**

This study demonstrated the capability of intra-fractionated PET imaging and radioactivity range measurement and will enable the investigation on the feasibility of intra-fractionated, range-shift compensated adaptive proton therapy.

## 1. Introduction

Protons and other charged particles can stop sharply inside the tissue and deposit the high radiation dose within the narrow Bragg peak at the end of a fixed depth (Kraft 2000, Smith 2006, Schulz-Ertner and Tsujii 2007, Schardt *et al* 2010). This physical property allows proton therapy (PT) to selectively deliver the escalated radiation dose to the tumor while reducing the dose and toxic effects to normal tissues adjacent to the target volume, which potentially enables improved accuracy and flexibility in dose delivery for advanced applications (Kraft 2000, Delaney and Kooy 2008). The clinical advantage of PT has been demonstrated and it is applied in radiation oncology for multiple disease sites (Chan *et al* 2004, Mizoe *et al* 2004, Slater *et al* 2005, Zietman *et al* 2005, 2006, Miyamoto *et al* 2007, Fukumitsu *et al* 2009, Paganetti *et al* 2021). On the other hand, PT could be compromised and severe damage to normal tissues near the tumor might occur if proton beams were not delivered accurately. The *in-vivo* verification of proton beam range (BR) is critical for achieving accurate PT, reducing normal tissue damage and toxicity, and permitting to apply more effective treatment plans (Parodi *et al* 2007a, Knopf and Lomax 2013).

Among the methods of applying *in-vivo* imaging to verify proton BR, positron emission tomography (PET) has been investigated and applied in clinics (Parodi *et al* 2002, 2007b, 2008, 2023, Hsi *et al* 2009, Vecchio *et al* 2009, Zhu *et al* 2011, Tashima *et al* 2016, 2021, Bisogni *et al* 2017, Buitenhuis *et al* 2017, Yoshida *et al* 2017, Ferrero *et al* 2018, Parodi and Polf 2018, Pennazio *et al* 2018, Mohammadi *et al* 2019, 2020, 2022, Parodi 2020, Kraan *et al* 2022, Moglioni *et al* 2022, Toramatsu *et al* 2022, Cuccagna *et al* 2024). It images the distribution of proton-induced positron emission radioisotopes and measures the positron activity range (AR). The measured AR can be used to calculate BR based on their correlations (Parodi *et al* 2007b, Paganetti 2012). On the other hand, the measured AR can be directly compared to the expected AR that can also be calculated based on the expected BR, and the difference between the expected and measured ARs which is referred as the range shift in this paper can be used to assess the accuracy of the beam delivery and, if it is necessary, to improve PT accuracy with a range-guided adaptive proton therapy (RGAPT) to compensate the range shift.

There are two major approaches in terms of when the range is measured and RGAPT is applied: (1) inter-fractionated RGAPT that measures the range after each fractionated session although data can be acquired during or after the session and if necessary revise the treatment plan (TP) for the next fractionated session, and (2) intra-fractionated RGAPT that measures the range and if necessary revises TP and deliveries the modified remaining beams all within the same session. Technically, intra-fractionated RGAPT is advantageous as it measures and corrects a possible range shift during the same session to avoid the potential change of proton BRs between the two sessions that can be caused from the uncertainty and variation of beam delivery, treatment positioning and alignment, patient physiological and tissue density changes after a treatment, and body and internal organ motion, etc. However, practically, it is challenging to achieve intra-fractionated RGAPT as it requires to measure the AR and complete the TP revision on-line without increasing the overall dose and dose distribution nor extending the overall session time substantially.

We have previously proposed an intra-fractionated RGAPT method and investigated its feasibility with algorithm, simulation, and experiment studies (Shao *et al* 2014, Chen *et al* 2018, Zhong *et al* 2020). Basically, the method will use part of the therapeutic beams to measure the AR while still be capable to revise the rest beam deliveries for compensating the possible range shift and delivering the overall dose and distribution as planned. To both minimize the radiation risk to the normal tissue beyond the targeted distal falloff boundary and have sufficient dose for improving the PET imaging count, the method uniquely selects an initial beam (or multiple beams) from the planned therapeutic beams with its Bragg peak at around the middle of the target region (mid-range for short) as a probing beam for the imaging and AR measurement, while the number of spills, intensity, and mid-range position will be specific PT plan and PET imaging capability dependent (Chen *et al* 2018). As shown in these previous studies that the method can have the combined strength and advantage from integrated radiation beam selection and on-line imaging to increase the image count to significantly enhance the PET image quality and range measurement accuracy, conserve the overall dose distribution, and minimize the risk of overshooting to a critical organ at risk (Zhong *et al* 2020).

We have recently developed a prototype human brain PET, evaluated its imaging performance and capability for on-line AR measurement with low-dose proton radiation, and experimentally conducted proton irradiated clinically realistic phantom studies to investigate the feasibility of achieving intra-fractionated RGAPT with the new method. As the first part, this paper reports the PET development and evaluation of its imaging performance and capability for on-line AR measurement. The feasibility study on achieving intra-fractionated RGAPT will be reported in the second part.

## 2. Materials and methods

### 2.1. PET for on-line imaging

The prototype PET was designed and developed for the fast data acquisition of proton-induced positron-emitter radioactivity range within a human brain with sufficient spatial resolution and sensitivity and operated in a clinical radiation environment. The following describes the major components of the PET system.

#### 2.1.1. Detector

**Figure 1.**
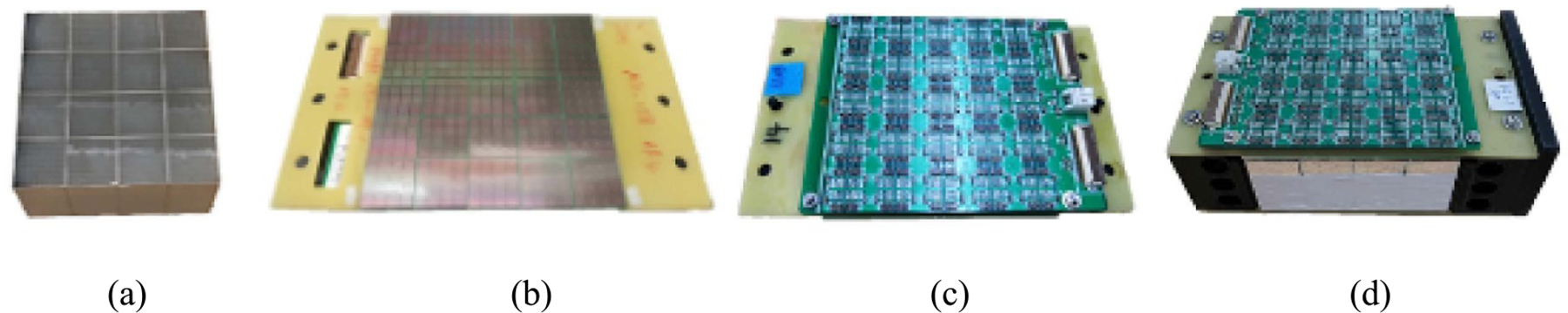
Photos of (a) a panel of 32 × 32 LYSO scintillators, (b) a panel of 5 × 5 SiPM arrays connected on one side of a PCB, (c) a resistor-based signal multiplexing readout board connected on the other side of the PCB, and (d) an assembled detector with mechanical fixtures to firmly hold all detector components together.

As shown in figure 1(a), each detector has a flat-panel of 32 × 32 array of 2 Lutetium–Yttrium 2 × 30 mm^3^ Ce-doped Oxyorthosilicate (Lu_0.6_Y_1.4_SiO_0.5_:Ce, LYSO) scintillators. All four 2 × 30 mm^2^ long surfaces of each scintillator were previously lapped with around 0.03 mm grade for depth-of-interaction (DOI) measurement while the two 2 × 2 mm^2^ end surfaces were mirror polished. Each scintillator is optically isolated from other scintillators with 0.06 mm reflective films (ESR, 3M Corp) between them to prevent inter-scintillator optical cross-talks. All outside surfaces of the scintillator array, except the one connecting to the silicon photomultiplier (SiPM) arrays, were wrapped with the ESR reflective films and white Teflon tapes to improve the scintillation light collection. The overall active volume of each detector is around 66 × 66 × 30 mm^3^.

One end of the scintillator array is optically connected to a panel of 5 × 5 array of multi-pixel SiPM arrays (MPPC S12642–0404PB-50(x), Hamamatsu Photonics, K.K.). Each SiPM array has 4 × 4 pixels, with 3 × 3 mm^2^ individual pixel size, <0.2 mm insensitive edge between pixels, and overall 13.3 × 13.3 mm^2^ array size. Each SiPM pixel has around 3000 micro cells, with a nominal gain of 1.25 × 10^6^ at the operating voltage around 67V. As shown in figures 1(b) and (c), all SiPM arrays within each panel were seamlessly tiled together with the overall size of around 66.5 × 66.5 mm^2^ that perfectly matches to the scintillator array size. Each panel has total 400 individual SiPM pixels.

Figure 1(c) shows an additional signal multiplexing board was connected on the other side of the printed circuit board (PCB) to reduce the total 400 anodes and 400 cathodes of individual SiPM pixel outputs to only 20 row (anode) and 20 column (cathode) detector-level signal output channels, which is based on the method of a resistor-based symmetric charge division (Wang *et al* 2016).

Figure 1(d) shows an assembled detector. All detector components were compactly held together with the PCB and 3D-printed plastic low-density mechanical holders on two sides of the detector but leaving the other two sides open so that neighboring detectors can be tightly placed next to each other for assembling a closely packed detector ring.

#### 2.1.2. Detector-level readout and process electronics

The recently developed compact 96-channel detector front-end readout and process electronics was used for this PET application (Cheng *et al* 2019). As shown in figures 3 and 4, each detector-level electronics board include an analog signal readout board and a digital board that has an onboard FPGA (Cyclone V 5CEBA7, INTEL Inc.) for interaction signal and event data processing. It includes a dual-polarity sigma-delta circuit and carry-line based FPGA time-digital-convertor with online self-calibration ability and ~70 ps (RMS) intrinsic timing resolution (Wang *et al* 2015, 2017). The maximal front-end electronics data processing rate for each detector panel is 250 K s^*−*1^ that is determined by the 4 *μ*s discharging time of the sigma-delta circuit that contains a charging stage to accumulate the signal charge and a FGPA-controlled discharging stage to digitally count the charge. The 4 *μ*s discharging time was selected based on the tradeoff of the data processing rate and the charge counting accuracy. A mini Display Port (miniDP) cable connects the front-end electronics of a detector panel to the data processing and acquisition electronics system board. A synchronization signal from system electronics to each detector provides 400 Mbpslow-voltage-differential-signals (LVDS) for data transmission from each detector to system electronics. Each detector-level electronics board can process maximum 96 channels input signals so that it was used to process signals from two detectors with total 40 rows and 40 columns of detector-level output signals. The size of each board is 84 × 84 × 1.6 cm^3^. A mini-size air fan is also attached to each board for temperature stabilization.

The output of each digitized detected singles event includes the measured event energy, timing, and position from the detected interaction signals. The event energy is the sum of all signals; timing is picked off with leading edge discriminator from the signal with maximum amplitude and corrected with energy-based time walk error correction.

Ten compact power supply boards were also developed to provide ±5 V to total twenty detector-level electronics boards and bias voltages to SiPM arrays. The bias voltage from each power supply board can be independently adjusted for optimized individual detector performance.

#### 2.1.3. Gantry

**Figure 2.**
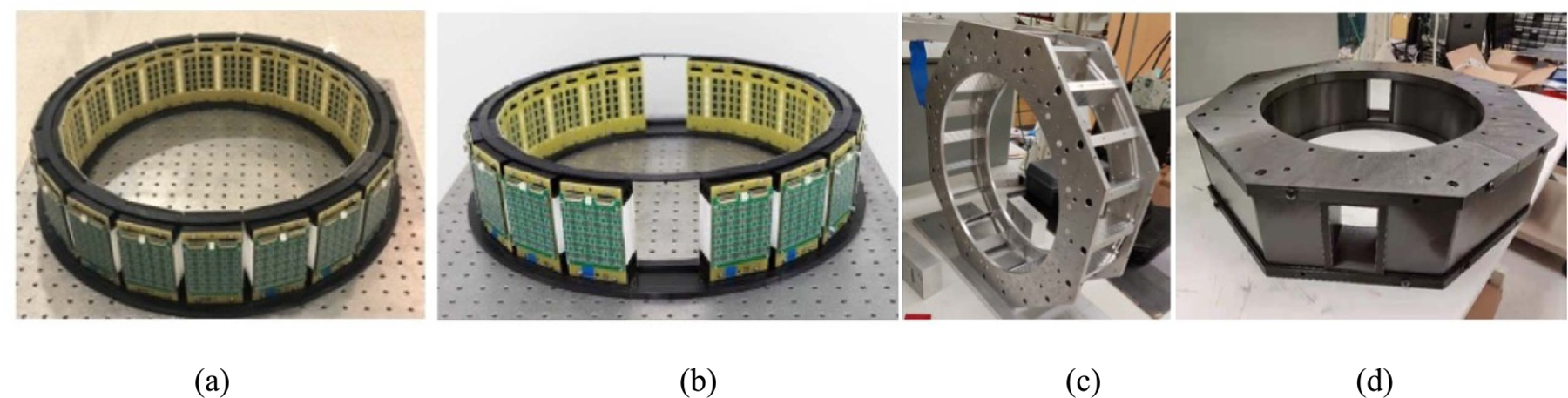
Photos of (a) a closed-ring PET configuration with 20 detectors, (b) an open-ring PET configuration with 18 detectors, (c) a metal gantry to insert all detectors steadily and precisely in their positions, and (d) the assembled PET in open-ring configuration with two open entrances and external light shielding cover.

As shown in figures 2(a) and (b), the prototype brain size PET can be configured as either a closed-ring configuration with 20 detectors in a multi-polygon shape for conventional full tomographic PET radiotracer imaging or an open-ring configuration with 18 detectors for proton-induced positron-emitter imaging. In the open-ring configuration, two detectors along the incoming proton beam axis are removed for passing proton beams through the PET from either direction to irradiate the target. The inner diameter of the detector ring measured between the two opposite detectors is around 41.8 cm and the height of the detector ring measured with covered scintillators is around 6.6 cm.

As shown in figure 2(c), a metal gantry was fabricated for inserting all detectors precisely and steadily at their designed positions inside the gantry. This metal gantry also prevents the scattered charge particles generated from proton interacting with the sensitive SiPM and readout and processing electronics that are behind the scintillator arrays. The slots around the gantry face were used to pass through thin flex cables for voltage supply and signal transfer that connect the detectors and the readout and processing electronics boards outside the gantry. Figure 2(d) shows all outer sides of the gantry were enclosed by 3–5 mm thick 3D-printed carbon-fiber sheets with various shapes and curvatures to light-tight shield the detectors. It has an octagon outside shape and two 10.0 × 5.4 cm^2^ open entrances for passing proton beams. PET can stand steadily on a flat surface and be rotated if needed for pointing the open entrances to a horizontal, vertical, or a beam with 45-degree angle between the horizontal and vertical angles. The distance from one octagon side to the opposite side is 55 cm. The overall imaging port is about 38.9 cm diameter and 14.9 cm axial length. The weight of the PET is about 40 kg. The imaging field of volume (FOV) is 32 cm diameter (in-plane) and 6.5 cm axially.

#### 2.1.4. System electronics and data acquisition

**Figure 3.**
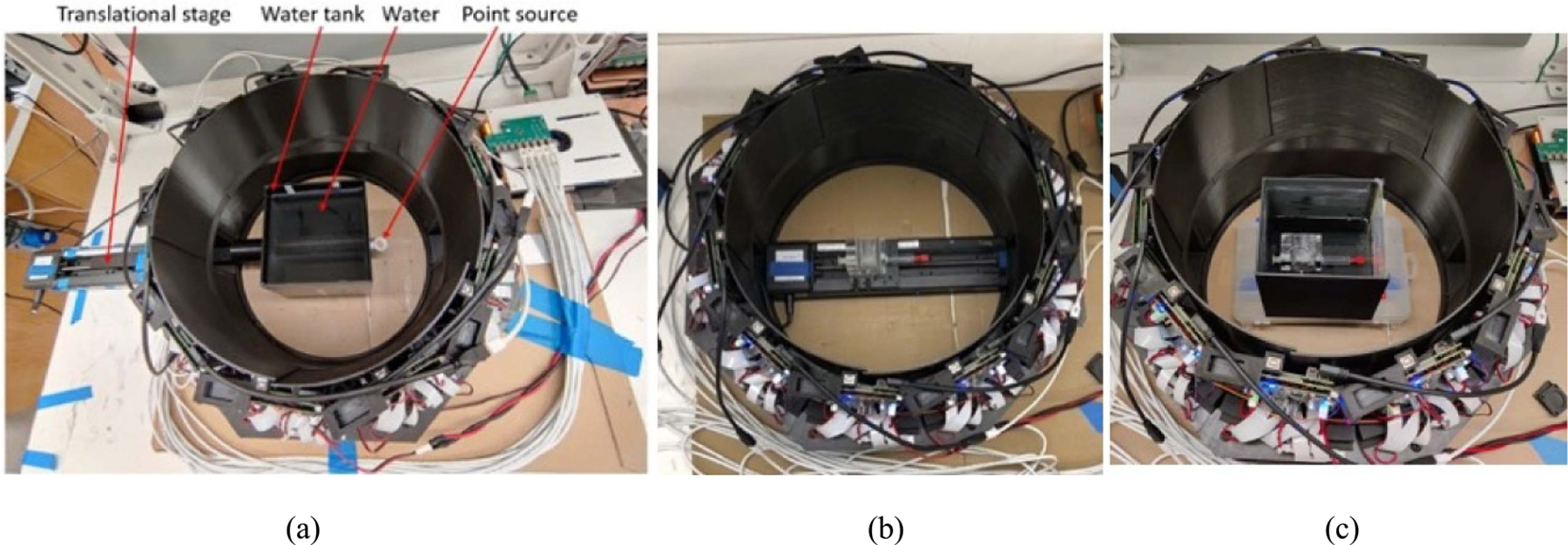
Photos of (a) a moving ^22^Na point source within a water tank for assessing the positioning accuracy from PET images with and without filling the water inside the tank, (b) a moving cylindrical phantom filled with ^18^F radioactive source for measuring the activity range from the corresponding distal falloff edge of the image profile, and (c) the same cylindrical phantom inside a water tank for assessing the impact of attenuation and scatter events to the accuracy of activity range measurement.

As shown in figure 3(a), all detector-level readout and processing electronics are attached to the backside of the gantry. Detected signals from multiplexing boards inside the gantry were transferred through flex cables to the readout and processing electronics boards and their digitized output signals were transferred through 10 2 m long LVDS cables to a FPGA board (TR4–230 Development Kit, Terasic Inc) based system electronics for coincidence events selection and other system-level signal processes. Distributed coincidence processors were installed at the system-level FPGA to independently and parallelly select and process coincidence events acquired within the FOV from total 35 detector pairs (Cheng *et al* 2021a). A PCIe link that can provide *~*20 Gbit s^*−*1^ maximal data transfer rate was used to transfer selected coincidence events to the computer (Cheng *et al* 2021b). System electronics and acquisition computer can be placed 1–2 m away from the PET gantry and proton interaction site to minimize the scattered charge particles and radiation photons.

#### 2.1.5. Basic system and imaging performance evaluation

**Figure 4.**
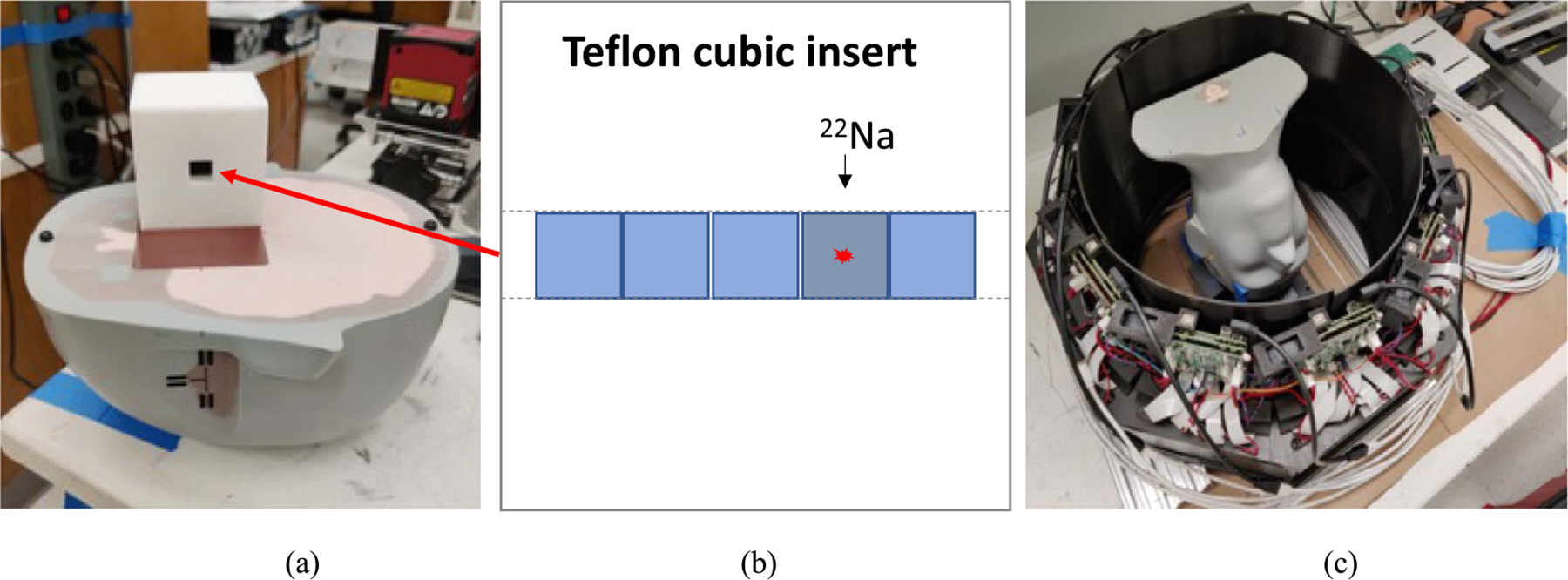
Photos of (a) the top of the STEEV phantom and a 3D printed cubic insert which has a rectangular hole in the middle, (b) a ^22^Na point source and the other four cubes were closely placed together and tightly inserted inside the hole of the cubic insert thus to ensure the point source was at a designated measured position, (c) the STEEV phantom was placed inside the PET with the point source within the imaging FOV.

A *~*700 KBq ^22^Na point source with *~*1.0 mm diameter radioactivity was positioned at the center of FOV (CFOV) to evaluate the detector crystal identification, energy and coincidence timing resolutions, and system sensitivity. For measuring the trans-axial spatial resolutions, the same point source was also placed on a linear translation stage and moved in the central axial FOV plane at 19 different radial positions from 90 to *−*90 mm off the CFOV with a 10 mm separation between the neighboring positions (figure 4(a)). Similarly, the axial spatial resolutions were measured with the point source moved along the system axis at 7 axial positions from *−*30 mm to 30 mm off the CFOV with a 10 mm separation between the neighboring positions. Minimal electronics threshold was set at around 30 mV through all data acquisitions. The data were acquired for 120 s at each FOV position with the closed-ring PET configuration while the results with the open-ring configuration were also obtained by removing the data associated with the two detectors to be removed in the open-ring configuration. Data were processed offline with a 350–650 KeV energy window and 20 ns coincidence timing window, with about 260 000 coincidence events at each FOV position being selected for image reconstruction. An open-source code (CASToR) was used for ordered subset expectation maximization based image reconstruction with 5 subsets and 10 iterations (Merlin *et al* 2018). No resolution recovery was applied in the reconstruction. A Gaussian function was fitted to each profile of the point source image and full width at half maximum (FWHM) was calculated as the spatial resolution at each FOV position.

### 2.2. Assessment of AR measurement capability with offline PET imaging study

The capability and performance of AR measurement by PET were initially assessed with radioactivity sources and phantoms in the lab. The study was focused on assessing the difference between individually measured and physical positions of the radioactive source at different FOV positions with and without attenuation and scatter events, and the accuracy of AR measurement with a distal falloff edge of image profiles acquired from a known PET radiotracer distribution that mimicked the proton-induced positron-emitter radioactivity distribution.

#### 2.2.1. Accuracy of spatial positioning with PET imaging of a ^22^Na point source

As shown in figure 3(a), the same *~*700 KBq ^22^Na point source was placed inside a water tank filled with 16 × 16 × 16 cm^3^ water. The point source was moved in the central axial plane from *−*100.0 mm to 100.0 mm off the CFOV with 10.0 mm each step and coincidence data were acquired at each FOV position for 120 s. Entire experiment was conducted twice with or without water in the tank, and the corresponding measured point source position as the center of the reconstructed image and the physical position of the source were compared for each FOV position where data were acquired.

Additionally, a Stereotactic End-to-End Verification (STEEV) phantom (Model 038, CIRS, Norfolk, VA, USA), which is an anthropomorphic human head phantom to check the necessary steps in radiotherapy treatment planning system from diagnostic imaging with CT, MR or PET to treatment plan verification, was used to evaluate the accuracy of the spatial positioning with PET image. As shown in figure 4(a), the bottom part of the phantom allows to insert an interchangeable cube for different imaging and radiotherapy study. A 60 × 60 × 60 mm^3^ cubic insert with solid Teflon material was fabricated by 3D printing, with a 10 × 10 × 60 mm^3^ hole through the center of the insert. A ^22^Na point source with 251 kBq activity [MMS03 Multimodal Imaging Source, Eckert & Ziegler Isotope Products, USA] was inserted inside the hole of the Teflon cubic insert. The physical ^22^Na radioactivity material is sealed at the center of a plastic cube with 10 × 10 × 10 mm^3^ volume. The spherical radioactivity source with around 0.25 mm diameter is precisely at the geometric center of the cube. To ensure the point source will be accurately positioned at a specified position inside the cubic insert, four plastic cubes with the same 10 × 10 × 10 mm^3^ volume were also 3D printed and inserted inside the cubic insert, as shown in figure 4(b). Depending on where the point source was placed along with the other cubes, the point source can be placed accurately at five specified positions within the cubic insert. Figure 4(c) shows that after the cubic insert was inserted inside the bottom of the phantom, which was affixed steadily inside the PET FOV, the top of the phantom was aligned and attached to the bottom of the phantom for imaging. For imaging the point source at a different FOV position, the top of the phantom was removed first, the cubic insert removed next, and the position of the cube containing the source interchanged with one of plastic cubes, and the cubic insert and the top of the phantom were sequentially placed back again. All involved components were accurately fabricated and experiment mechanical processes were fixed and tested for good imaging stability and repeatability.

Total five images at different FOV positions were acquired within the phantom, with 10 mm distance between neighboring positions. An additional image with the point source at the known external phantom edge was also acquired as the reference, with a 74.5 mm distance between this source position and the central source position inside the phantom. Acquisition time at each source position was 90 s. No attenuation and scatter corrections were applied which is required in the fast on-line PET imaging and intra-fractionated AR measurement.

#### 2.2.2. Accuracy of AR measurement with PET imaging of a known radioactivity distribution

For assessing the capability of AR measurement, a syringe with a flat end surface was used as a phantom to provide a cylindrical radioactivity distribution with a sharp distal falloff edge. As shown in figure 3(b), the syringe filled with ^18^F radioactivity was placed on the linear translation stage and moved within the central axial plane along the horizontal axis with a 0.5 mm step between each two sequential acquisition positions. The activity intensity was around 685.1 KBq/cc at the beginning of the study. Coincidence events were acquired at each position for 60 s, and each dataset was divided into 9 equal count subsets for calculating the mean and standard deviation. The image was reconstructed with 1 subset and 20 iterations. The profile across each image center along the beam direction was selected to display the image intensity distribution, and the position at the 50% distal peak of each intensity profile was calculated as the distal falloff edge position and was also used to further calculate the AR.

To investigate the impact of attenuation and scatter effect to the distal falloff edge position and AR measurement, the syringe was placed inside the water tank (figure 3(c)). Images with and without the water filled inside the tank were acquired, and the corresponding image profiles and ARs were calculated and compared. This comparison study was conducted for one syringe position.

### 2.3. Assessment of AR measurement capability with on-line PET imaging study

The capability of PET image-based AR measurement was investigated at a PT center with pencil scanning beams and two different phantoms. The study focused on understanding impacts to the accuracy and precision of AR measurement from major factors that include the attenuation and scatter events, LYSO intrinsic background, and proton dose.

#### 2.3.1. Study with a uniform Lucite phantom

**Figure 5.**
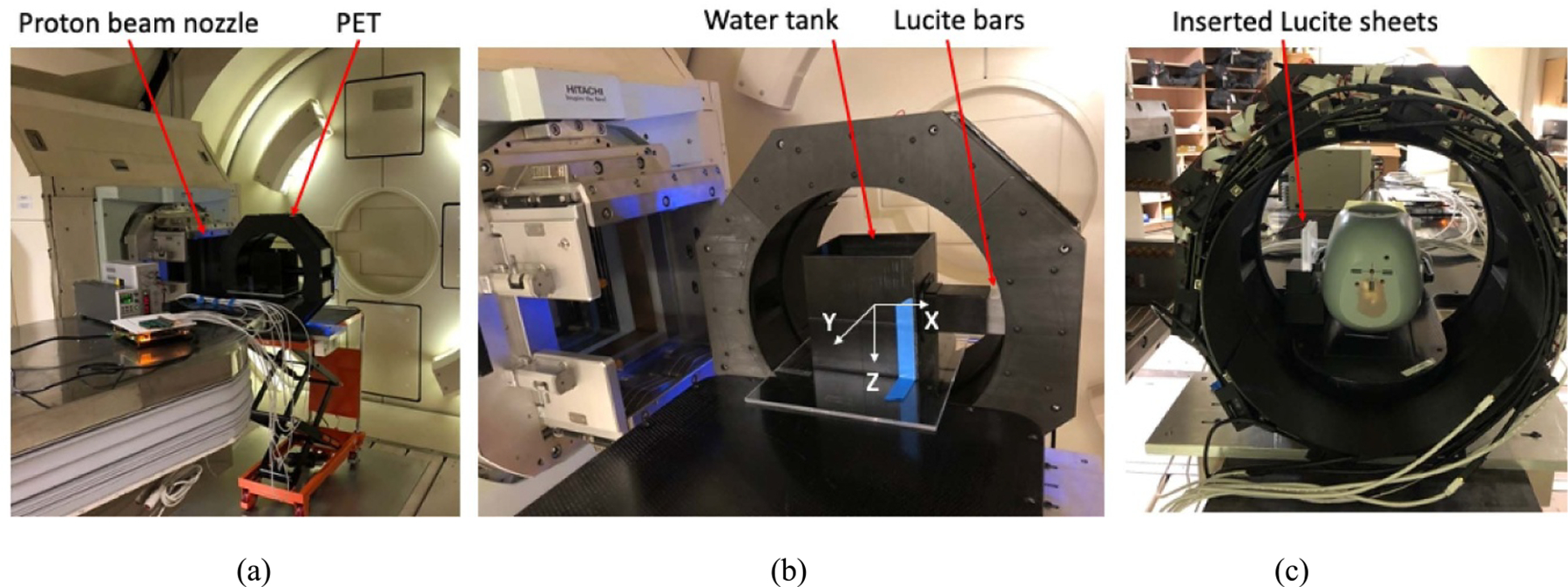
Photos of (a) experiment setup with the PET on a mobile lifting cart and aligned to the proton beam nozzle, (b) two Lucite bars were stacked together and inserted inside a water tank for proton radiations, with the direction of incoming proton beams parallel to the PET *x*-axis, and (c) the setup with a STEEV phantom as viewing from the other side of the PET.

The experiment setup is shown in figure 5. PET was placed on a mobile lift cart with its open entrance aligned to the proton beam nozzle. Power supplies and system electronics were placed more than a meter away from the PET to minimize the scattered radiations. Two rectangular Lucite bars, each with 2.5 × 5.1 × 35 cm^3^ volume, were stacked together as a uniform phantom and inserted into a water tank filled with 16 × 16 × 16 cm^3^ water for most studies except it was emptied for the study without attenuation and scatter effects. A Gafchromic film (Gafchromic EBT3, Ashland Inc.) was inserted between the two bars for recording the proton interactions as a reference to compare the AR measured by PET.

Protons with pristine energy and *~*9 mm beam radius (spot beam) irradiated the Lucite phantom from the 2.5 × 5.1 cm^2^ side. PET acquisition started immediately after the completion of the beam delivery for 300 s while different subsets of data were selected offline for different studies, such as the first 60 s data were selected for most AR measurement studies. Acquired data were selected with 350–650 KeV energy and 20 ns coincidence timing windows but without attenuation and scatter data corrections. The PET imaging coordinate is also shown in figure 5(b). Images were reconstructed by CASToR with 1 subsets and 10 iterations. The image slices in *x–z* (coronal) plane and within the beam central region with similar intensities were combined for obtaining sufficient count statistics and the corresponding intensity profile was drawn and displayed for activity distribution analysis and AR measurement.

Different number of Lucite sheets were inserted between the beam nozzle and the Lucite phantom for introducing different range shifts. Each Lucite sheet has 6.5 × 6.5 cm^2^ size and 6.4 mm physical thickness and was placed with its 6.5 × 6.5 cm^2^ planar surface perpendicular to the beam direction. With 0, 1, or 2 inserted Lucite sheets, beams with overshot, planned, or undershot BRs were introduced for various range-shift measurement studies.

#### 2.3.2. Study with STEEV phantom

The imaging and AR measurement studies were also conducted with STEEV phantom under the same radiation and imaging setup. Gafchromic films were cut and inserted inside the cubic insert. As shown in figure 5(c), Lucite sheets were also inserted for range-shift related studies. The proton irradiation, data acquisition, image and data processing, and AR measurement were very similar as the study with the Lucite phantom.

The impact of LYSO intrinsic radiations from ^174^Lu was also investigated. A 60 s PET dataset was acquired after single-spot beam protons with 109.4 MeV energy and 25 MU dose irradiated STEEV phantom. A 1/25 of acquired full dataset was evenly sampled over the 60 s PET acquisition time to generates 25 sub datasets with each sub dataset has the equivalent 1 MU dose radiation and only 1/25 s background events. Such sub dataset is referred as the minimal-background-radiation sub dataset. Additionally, a 60 s PET blank scan without any radioactivity within the FOV was separately acquired, and different fractions of this background random event data were added to one minimal-background-radiation sub dataset for measuring the corresponding ARs with different levels of background noise to study the impact of background random events to the AR measurement. The same 350–650 KeV energy and 20 ns coincidence timing windows were applied in this study.

## 3. Results

### 3.1. PET detector and system performance

**Figure 6.**
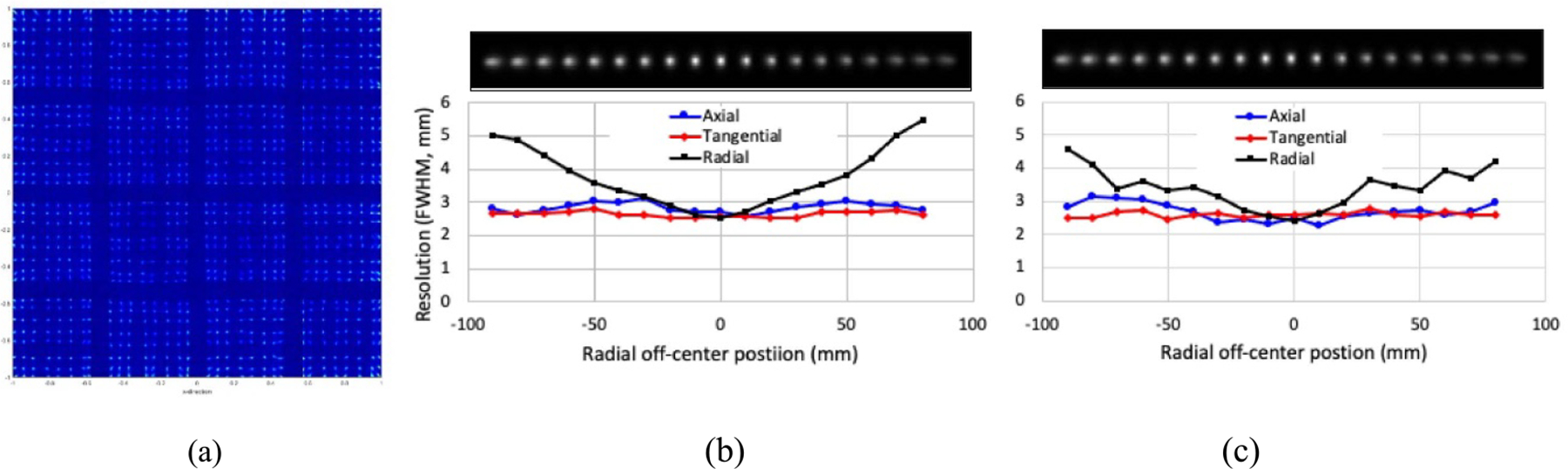
(a) Measured crystal intensity map, and reconstructed images and measured spatial resolutions with (b) close-ring and (c) open-ring PET configurations.

As shown in figure 6(a), all 1024 LYSO scintillators in a detector can be clearly separated for crystal identification. The mean energy and coincidence timing resolutions measured at the FWHM were ~27.2% and *~*9.2 ns respectively (Yang *et al* 2021).

Figures 6(b) and (c) show the reconstructed images of the ^22^Na point source at different radial off-center FOV positions and the corresponding spatial resolutions measured with close-ring and open-ring PET configurations. The difference in spatial resolutions between the two configurations at each measured position was <1.0 mm, with the mean and standard deviations being 0.18 mm and 0.34 mm respectively. The sensitivity at different radial FOV positions was also measured directly from the same acquired dataset. Above 3% sensitivity was achieved over the FOV with either close-ring or open-ring configuration.

### 3.2. AR measurement capability assessed with offline PET imaging

**Figure 7.**
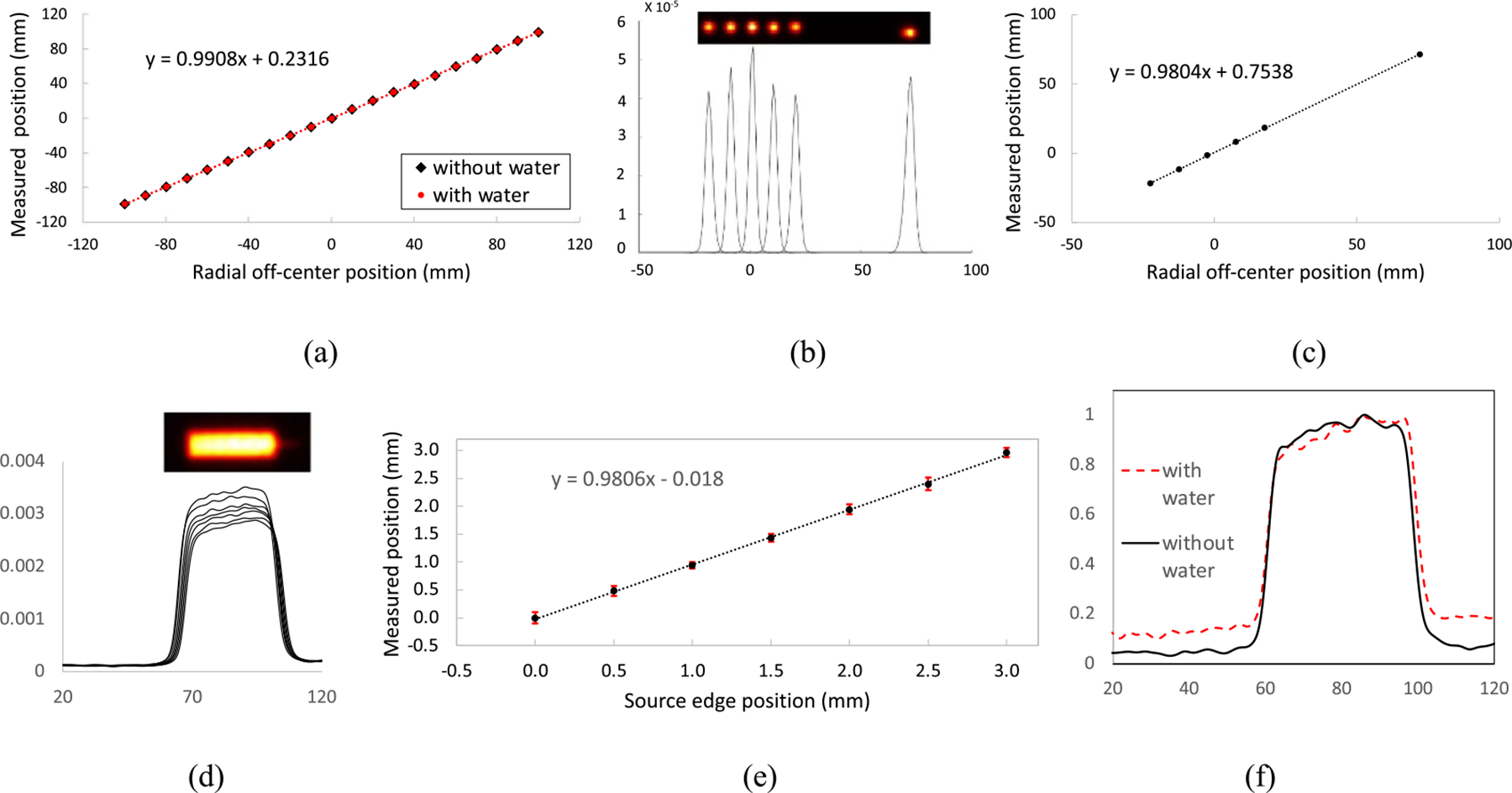
(a) Linear relationship between the measured and physical positions of the ^22^Na point source across the FOV in air, (b) radial profiles of reconstructed point source images over radial FOV positions measured with the STEEV phantom and (c) their corresponding linear relationship between the measured and physical positions of the source. (d) One reconstructed cylindrical phantom image and profiles of such images acquired with the phantom at different positions, (e) linear relationship between the measured distal edge positions and the phantom physical positions, and (f) normalized profiles of images acquired with and without attenuation and scatter events.

Figure 7(a) shows a linear relationship between the measured and physical peak positions of radial profiles drawn from the reconstructed images of the ^22^Na point source at different radial off-center FOV positions with or without the attenuation and scatter effects from the water in the water tank.

Figures 7(b) and (c) show the similar measured source positioning and linearity from the STEEV phantom study as described in figure 4. The measured ^22^Na point source positions were well matched with the physical source positions within the phantom. These results indicate that the PET image can accurately measure the concentrated radioactivity position inside the phantom even with nonuniform geometry and material densities.

Figure 7(d) shows the reconstructed image acquired from the cylindrical phantom at one measurement position and the intensity profiles drawn from images at seven different measurement positions. The level of intensity was gradually reduced because of ^18^F isotope decay over the course of the study. The nonuniform intensity distributions were mainly due to the uncorrected minor sensitivity variation and attenuation changes from the phantom holder. Each distal edge position was calculated as the position where the intensity is the 50% of the distal peak which was defined as where it has the maximum curvature value. Figure 7(e) shows that the measured shift of distal edge positions well matched with the shift of physical phantom positions.

Figure 7(f) shows that although the impact of attenuation and scatter effects did reduce the acquired intensity of the profile, the distal edge can still be accurately measured. With the width of a profile being measured as the distance between the distal and proximal edge positions, the difference between the widths of the two profiles with or without the attenuation and scatter events from the water was merely 0.3 ± 0.3 mm.

### 3.3. AR measurement capability assessed with on-line PET imaging

#### 3.3.1. Impact of attenuation and scatter events

**Figure 8.**
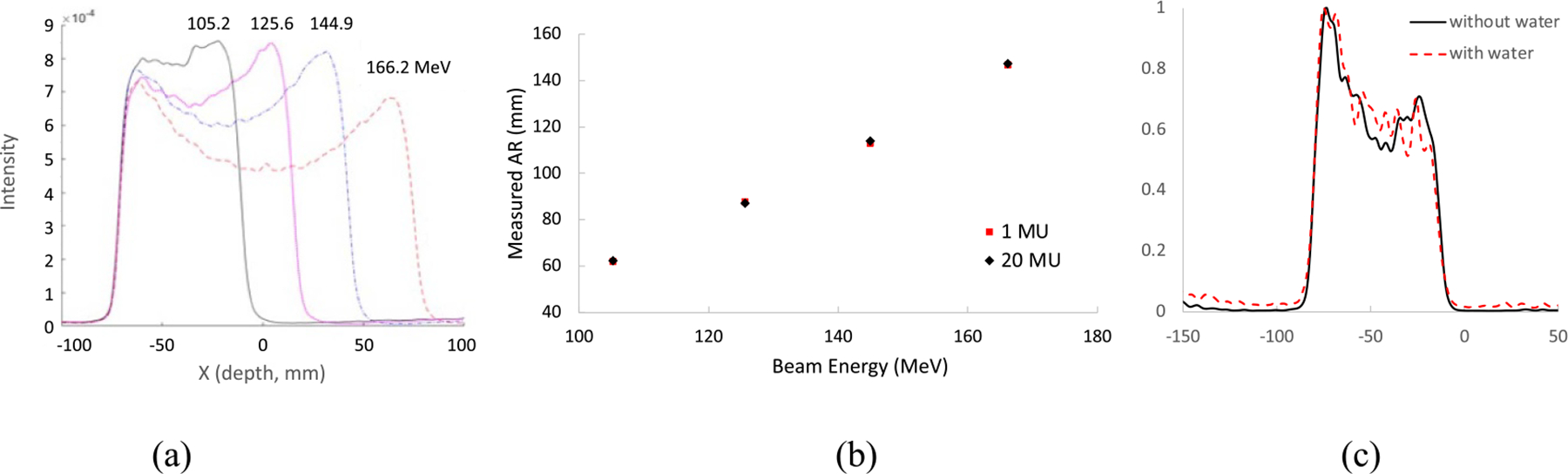
(a) Intensity profiles of reconstructed images with the data acquired from 105.2, 125.6, 144.9, and 166.2 MeV proton beams irradiated the Lucite phantom, (b) measured AR vs beam energy with 1 and 20 MU doses, and (c) normalized image intensity profiles with the data acquired with and without water in the water tank.

Figure 8(a) shows the intensity profiles of reconstructed images from the data acquired after single-spot proton beams with 20 MU dose and 105.2 MeV–166.2 MeV beam energies irradiated the Lucite phantom without attenuation and scatter events from the water tank. The distal falloff edges are smooth and AR can be reliably measured. Figure 8(b) shows that the AR can be measured with about the same accuracy at 20 MU or 1 MU proton dose if without attenuation and scatter events. Figure 8(c) shows the intensity profiles of reconstructed images from the acquired data after single-spot proton beams with 25 MU dose and 109.4 MeV energy irradiated the Lucite phantom with and without the attenuation and scatter events from the water tank. The total acquired counts were reduced by about 50% with the attenuation and scatter effects. However, both distal and proximal falloff shapes were about the same, so as their edge positions. The difference between their measured 50% distal falloff edge positions with and without attenuation and scatter effects was *~*0.2 mm.

#### 3.3.2. Impact of LYSO scintillator intrinsic radiation

**Figure 9.**
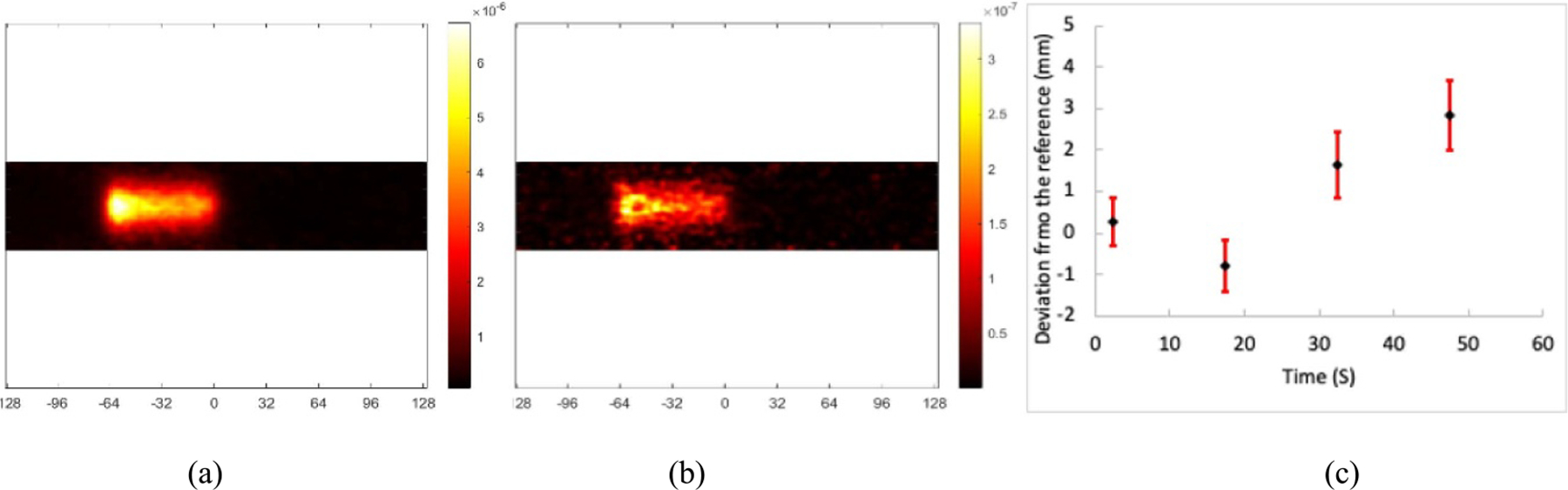
Images reconstructed with the data of (a) 25 MU dose and 60 s acquisition (reference), (b) equivalent 1 MU dose and 2.4 s blank acquisition, and (c) differences between the reference AR and the ARs measured with the equivalent 1 MU dose combined with different levels of background noises acquired from different acquisition times of the blank scan.

Figures 9(a) and (b) show the reconstructed images from the full dataset acquired after single-spot proton beams with 25 MU dose and 109.4 MeV energy irradiated STEEV phantom and a single minimal-background-radiation sub dataset with equivalent 1 MU dose. Figure 9(c) shows the difference between the AR values measured with 25 MU and 1 MU is <1.0 mm by adding <20 s acquisition of background random events (blank scan) to the minimal-background-radiation sub dataset. However, this difference was increased to >1.0 mm if adding >30 s acquisition of background random events, indicating that, with *~*1 MU low dose proton radiations, the increased background random events from LYSO intrinsic radiations can proportionally, adversely impact the accuracy of the AR measurement.

#### 3.3.3. Impact of proton dose

**Figure 10.**
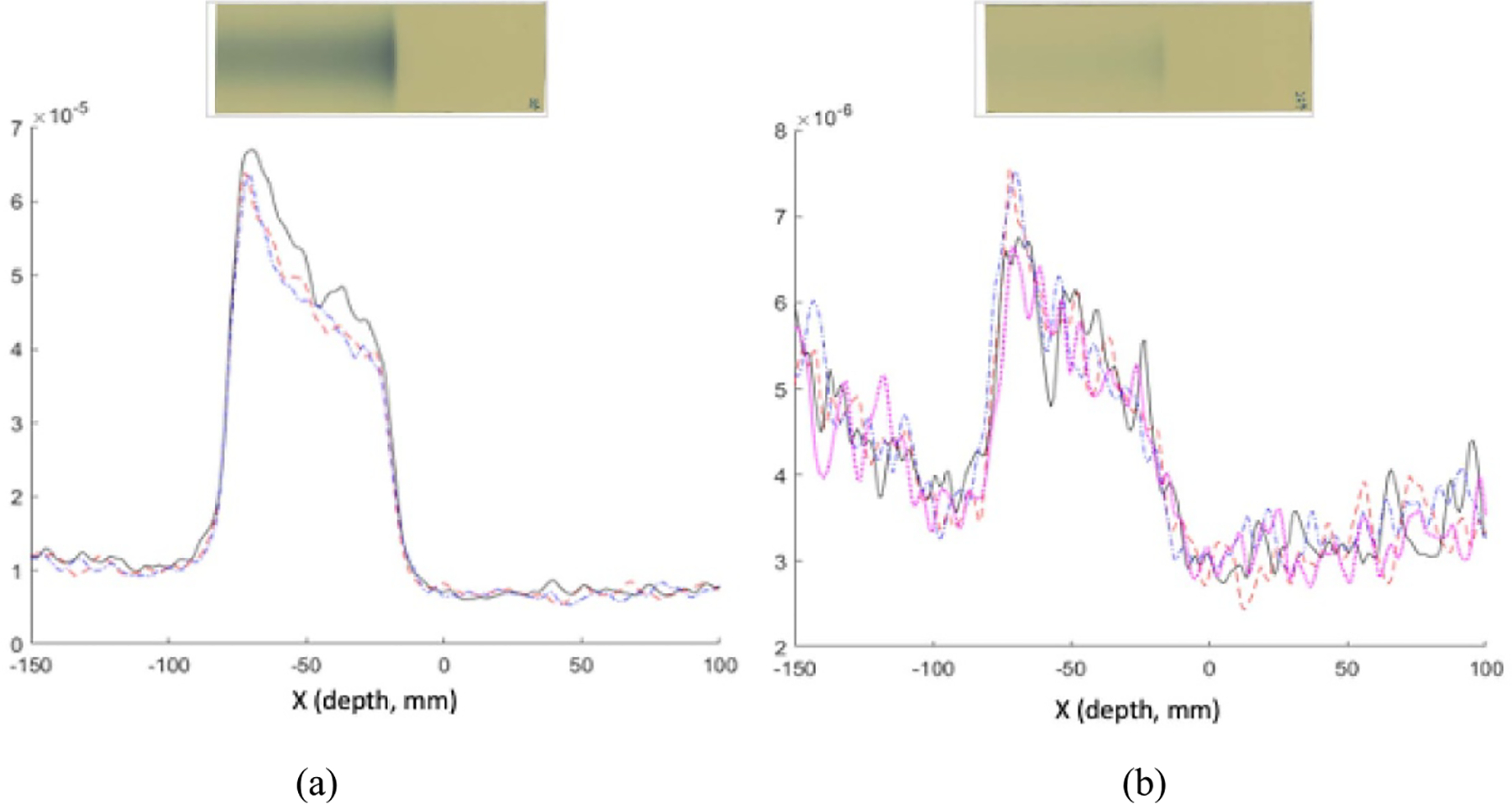
Recorded proton interaction film images and profiles of reconstructed PET images with three repeated irradiation-imaging studies and with (a) 10 MU and (b) 1 MU proton doses.

Figure 10 shows the recorded images of proton interactions by the film and the corresponding intensity profiles of reconstructed PET images from the acquired data of single-spot proton beams with 109.4 MeV energy and 10 MU and 1 MU doses repeatedly irradiated the Lucite phantom. As expected, the high dose irradiation provides not only higher signal-to-noise ratio but also the smaller variation among different measured proximal and distal edge positions. However, the measured means and standard deviations of ARs, which are the lengths between the proximal and distal edge positions, were about the same with 10 MU and 1 MU doses at the value of 59.9 ± 0.6 mm and 60.1 ± 1.0 mm, respectively, indicating that the proximal and distal edge positions were shifted about the same with 10 and 1 MU dose proton radiations.

**Figure 11.**
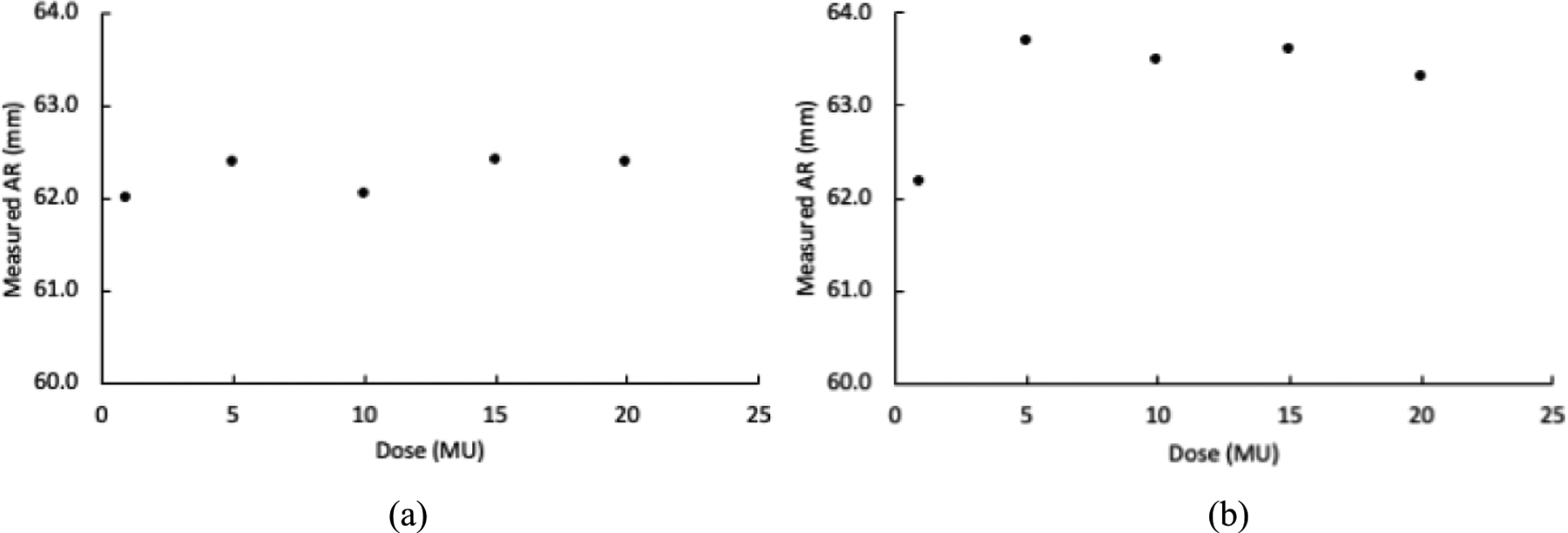
AR values measured from proton radiations of different doses with (a) Lucite and (b) STEEV phantoms.

Figure 11 shows the AR values measured from the acquired data of single-spot proton beams with 109.4 MeV energy and 1 MU to 20 MU doses irradiated the Lucite and STEEV phantoms. With Lucite phantom, the difference among all AR values measured from 1 MU to 20 MU doses were less than 0.5 mm. However, with STEEV phantom, the difference between AR values measured from 1 MU and the other 5–20 MU doses was more than 1.0 mm, indicating that the geometry and nonuniformity can deteriorate the accuracy of AR measurement with low dose proton radiations.

**Figure 12.**
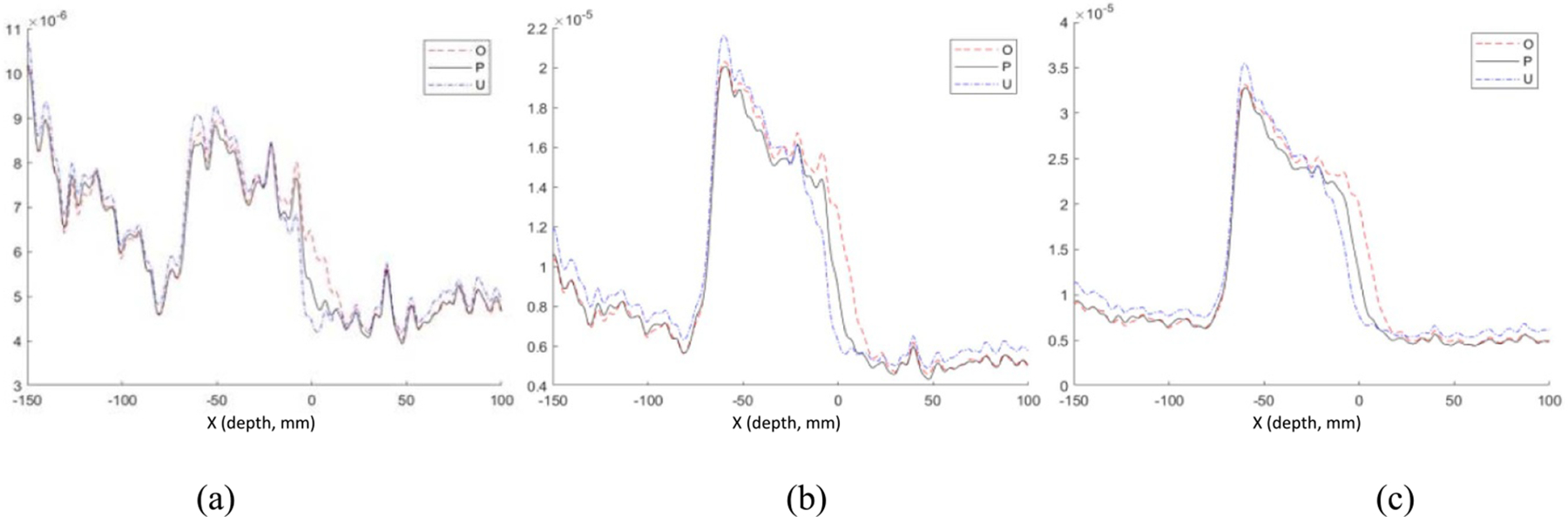
Plots of image intensity profiles measured from a single-spot proton beam with 109.4 MeV energy irradiated STEEV phantom with dose of (a) 1 MU, (b) 3 MU, and (c) 5 MU. With each dose, profiles were displayed in group with planned (P), overshoot (O), and undershoot (U) beams generated with different thicknesses of inserted Lucite sheet.

Figure 12 shows the image intensity profiles with three different ARs from the acquired data of single-spot proton beams with 109.4 MeV and 1, 3 and 5 MU doses irradiated STEEV phantom. The range shifts introduced by different number of inserted Lucite sheets can be well identified and measured with 5 MU dose proton radiations but not with the 1 and 3 MU dose radiations. The results of measured and expected AR shifts with 1–25 MU doses are summarized in table 1.

## 4. Discussion

This study has demonstrated the feasibility of intra-fractionated proton AR measurement based on-line PET imaging with 60 s acquisition time from a series of proton irradiated phantom studies. The results show that ~1 mm accurate AR measurement can be achieved even with moderate PET performance, limited counts of acquired proton-induced coincidence gamma rays, and without conventional attenuation and scatter data corrections.

The results also show that the intrinsic radiation from LYSO can have substantial adverse impact to the accuracy of the AR measurement at the low-dose proton radiation. It emphasizes the importance of having high coincidence time resolution that can reject more random events and further push the limit of minimal dose required for selecting a probing beam.

The flat-panel detectors and front-end readout electronics that were originally developed for a different research project were used for this research after retrofitting. The original detector design with reading out scintillation photons from both ends of a scintillator array (dual-scintillator-end readout) and roughed scintillator surface can provide the DOI measurement and overall good scintillation light collection efficiency (Sun *et al* 2015). However, the number of signal process channels and total PET development cost would be increased substantially for this project with the original design. The modification applied a single-scintillator-end readout and enhanced low-amplitude signal readout and processing electronics. The resulted detector performance of scintillator crystal identification, and energy and coincidence time resolutions were degraded moderately compared to that with the original design but still at the levels sufficient for this initial investigation on the feasibility of on-line proton AR measurement and RGAPT. On the other hand, if starting with an optimized detector design, the PET performance, particularly the coincidence time resolution, can be improved that would further improve the capability and accuracy of low-dose proton AR measurement and potentially achieve that with shorter than 60 s acquisition time.

The result has shown that lack of DOI measurement capability is not critical to the AR measurement, particularly for the measurement at near the CFOV, mainly because the distal falloff region is within a relatively small region so that the effect of spatial resolution nonuniformity with gradual spatial change is minor, especially when the required measurement accuracy is within 1–2 mm. On the other hand, the DOI measurement could become important in the future if multiple ARs across different FOV regions to be measured and compared for an advanced image guidance study.

At the low-dose proton radiation, the study has shown that the accuracy of AR measurement is determined by the effective count applied in the image reconstruction, while such counts is attributed by factors that mainly include the PET performance (e.g. sensitivity and coincidence time resolution), proton dose, geometry and material of the phantom, acquisition time and data selection, and AR measurement method. Among them, the proton dose and PET acquisition time are the dominate and most flexible parameters that are controllable for accurate AR measurement. However, in practice, they are still constrained by how high the dose of a single probing beam in a particular treatment plan can be and how long the overall process of intra-fractionated AR measurement and adaptively revised treatment plan will take. More than a single spot radiation can be selected as the probing beam. In this study, a single spot proton beam radiation and 60 s PET acquisition were chosen for initial investigation, however, in practice, a single layer or multiple adjacent single-layer beams can be possibly selected as combined probing beams to boost the overall dose and increase the count without jeopardizing the treatment replanning to compensate the range-shift.

The positron activity intensity profiles measured from the Lucite and STEEV phantoms appear distorted when no attenuation and scatter corrections were applied, such as the high counts measured at the entrance edges where there was no attenuation and scatter medium outside the edges. However, such distortions should not or very minorly affect the AR measurement accuracy since an AR is only measured within a narrow distal falloff region at the end the beam.

Positron range can also have adverse impact to the AR measurement accuracy, particularly in a patient study where higher concentration of proton-induced positron isotopes (such as ^15^O) with relatively large positron ranges could be produced. It is rather challenging to experimentally assess and compensate the impact of positron ranges to a gross AR measurement error. At this point, we view the impact of positron range to the AR measurement in our study is likely a less critical factor to consider when compared it to the other more critical factors such as proton dose, acquisition time, and count, etc.

The potential impact of neutron radiation to the PET performance was checked after each proton radiation study by measuring the detector energy spectrum and crystal intensity map. No systematic shifts of 511 KeV peak, background noise level, and crystal identifications were observed over the entire course of the study, and the variations among different measurements were sufficiently small without impacting the PET performance and AR measurement. This is mainly because the scanning proton beam has much lower level of neutron radiations that could potentially lead to severe PET performance changes.

The unit of proton beam intensity used in the radiation experiment measurements and data analyses was MU, which is a convenient and appropriate unit for this study that focused on investigating the PET image quality and AR measurement accuracy as a function of proton beam intensity. To estimate the level of radiation dose associated with proton beam intensity, we calibrated Gafchromic films that were exposed to the known proton radiations within the Lucite phantom, corrected relevant biases, and calculated the corresponding dose values (Casanova Borca *et al* 2013). The estimated radiation dose value for a 1 MU proton beam intensity was around 0.1–0.3 Gy with different phantom setup and experiment conditions (Yang *et al* 2022). It indicates that the proposed low-dose on-line PET imaging and AR measurement is feasible for a practical proton beam therapy.

In practice, the on-line PET coordinate can be registered to the treatment system coordinate by dual imaging (PET and x-ray) markers to directly allocate and register their spots within the two coordinates. This will also ensure the images between the PET and treatment plan are accurately aligned, given that the planning CT positions in the treatment coordinate are readily available through patient setup alignment in the treatment system. In principle, it is a one-time procedure to align the PET and treatment plan coordinates, and its repeatability can be warranted by placing the PET each time on the same position and orientation and be checked by lasers equipped with the treatment system. Therefore, it is also feasible to verify PET-measured AR based on the co-registered on-line PET and planning CT patient images, and to compare the measured and predicted AR images to assess the range shift.

It is expected to be more challenging to achieve the same accurate AR measurement in a clinical study with the same proton radiation dose and PET acquisition time used for the phantom study because of more adverse factors to be involved, such as greater tissue heterogeneity, more proton-induced positron radioisotopes with different intensities, distributions and positron ranges, and the addition of biological decay and washout effects. To compensate those adverse impacts without substantially increasing the proton dose and PET acquisition time, we can increase the prototype PET axial length to augment its axial FOV and sensitivity and substantially improve the current coincidence time resolution, which could lead to 2-to-3-fold improved count ability and be sufficient to provide the required compensation.

Although the initial investigation is based on a brain PET imaging, the approach is potentially feasible for a whole-body PET imaging application even with extra attenuation and scatter effects. Some PET modification and enhancement for AR measurement are expected but the basic technology and method should be the same or similar.

## 5. Conclusion

The feasibility study of achieving an accurate on-line proton AR measurement for intra-fractionated and range-shift compensated adaptive PT has been investigated. A dedicated PET was developed and evaluated for its imaging performance and capability for proton AR measurement, and various phantom experiments with proton irradiations were conducted to assess the accuracy of the on-line AR measurement under different radiation and imaging conditions. The initial study has shown that the PET is capable to acquire proton-induced coincidence gamma rays under PT environment and demonstrated that the accuracy and precision of the measured AR can achieve 0.6 ± 0.3 mm with a short acquisition time (60 s) and at a relatively low-dose (5 MU) proton beam. These results indicate the feasibility to select fraction of one or few proton beams from a practical proton treatment plan to accurately measure the AR and to potentially enable an intra-fractionated adaptive PT to fully compensate the range shift. Utilization of such technology might allow for a significant reduction in margins typically added to account for uncertainties in clinical treatments. Studies along this line will be reported separately.

## Figures and Tables

**Table 1.** AR shift as a function of proton dose.

Dose (MU)	AR Shift (mm)			
	Overshot		Undershot	
	Measured	Expected	Measured	Expected
1	9.6	6.8	−4.9	−6.8
3	7.6		−6.0	
5	7.1		−6.3	
6	7.0		−6.3	
25	6.8		−6.9	

## Data Availability

All data that support the findings of this study are included within the article (and any supplementary information files).
